# The high larval burden of *Trichinella britovi* in wild boar in Serbia

**DOI:** 10.2478/helm-2025-0027

**Published:** 2025-11-26

**Authors:** M. Dmitric, N. Vaskovic, V. Kurćubić, K. Matović, S. ŽIvković, N. Karabasil

**Affiliations:** 1 Veterinary Specialized Institute Kraljevo, Zicka 34, 36000 Kraljevo, Serbia; 2 Faculty of Veterinary Medicine, University of Belgrade, Bulevar oslobodjenja 18, 11000 Belgrade, Serbia; 3 Faculty of Agronomy in Čačak, University of Kragujevac, Cara Dušana 34, 32000 Čačak, Serbia

**Keywords:** high larval burden, *Trichinella britovi*, Serbia, wildlife

## Abstract

During a routine analysis of a wild boar diaphragm, a significant infestation with *Trichinella* spp. was found, larvae were identified, with a larval burden of 767 larvae per gram (LPG) of muscle tissue. To our knowledge, this is the largest infection detected to date in Serbia. By employing a polymerase chain reaction (PCR), the *Trichinella britovi* was the only species identified. The high LPG levels found in this study suggest that game meat has not been examined for the presence of *Trichinella* spp. pose a significant risk to human health and could potentially lead to fatal outcomes. Furthermore, this finding confirms the importance of wild boars as a source of infection of *T. britovi* in Serbia.

## Introduction

*Trichinella* spp. are zoonotic nematodes distributed worldwide, except in Antarctica (Pozio, 2007 and 2016; Marin *et al*., 2023). Humans become infected after consuming raw or undercooked meat containing infective larvae (Gottstein *et al*., 2009). According to the available data, 13 taxa have been classified in the genus Trichinella so far (Marin *et al*., 2023), with two species, *T. spiralis* and *T. britovi*, identified in Serbia (Dmitric *etal*., 2017). Additionally, T. britovi has previously been confirmed in Serbia in dried wild boar meat associated with human trichinellosis (Dmitric *et al*., 2018). Wild boar meat and its products are among the most common sources of *Trichinella* infections in humans (Pozio, 2015; Rostami *et al*., 2017). Therefore, this type of food should only be consumed after proper testing for the presence of *Trichinella* larvae (Dmitric *et al*., 2018).

The objectives of this study were:

1. To emphasize that *Trichinella* spp. Infection in game meat can be extremely high, highlighting the importance of meat inspection, especially for game meat, for detecting this parasite.
2. To use the method of histopathological diagnosis to determine how the high infection of *Trichinella* larvae affects the muscle tissue of the host (wild boar).
3. To identify the species of *Trichinella* that has led to the high infection of wild boar meat.
4. To confirm the importance of wild boars as reservoirs of *Trichinella* spp. in the Republic of Serbia.

## Materials and Methods

### Sample

The wild boar (female, estimated age 6 – 18 months) was legally hunted on November 28, 2021, where a single diaphragm sample was examined.

### Artificial digestion

**Fig. 1.**
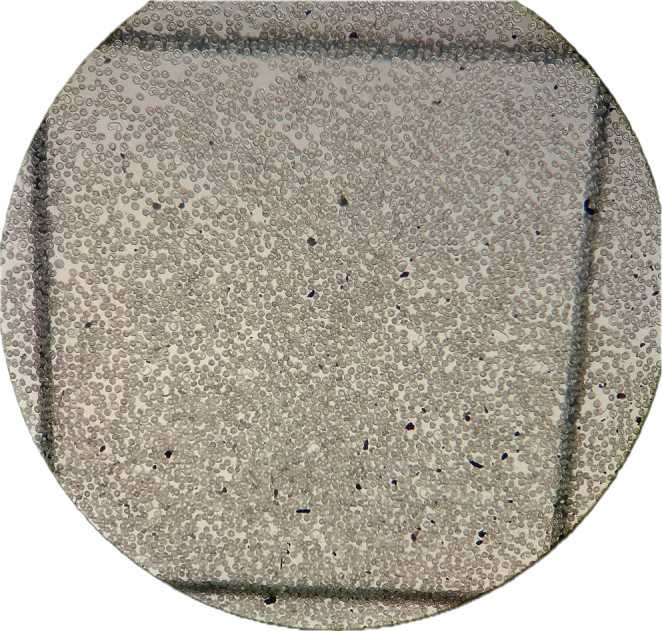
Microscopic findings after artificial digestion – *Trichinella* spp. (20× magnification)

A total of 10 g of muscle tissue was processed using the standard method “Microbiology of the food chain – Detection of *Trichinella* larvae in meat by artificial digestion method” (SRPS EN ISO 18743:2016). *Trichinella* larvae were enumerated using a microscope (MZ6 Stereo Microscope, Leica, Germany), and the infection levels were reported as the number of larvae per gram of sample (LPG) (Fig. 1).

### Histopathological examination

The sample was fixed in 10 % neutral buffered formalin. Following standard procedures in an automated tissue processor, the obtained sections were stained with hematoxylin and eosin (HE) and analyzed under a light microscope (BX51, Olympus Optical, Japan). The images were captured with an Olympus Color View III® digital camera.

### DNA extraction and amplification

After artificial digestion, the larvae were collected in tubes (Eppendorf Tubes 1.5ml) and stored in absolute alcohol at -20 °C until the beginning of the molecular confirmation of the *Trichinella* species. Deoxyribonucleic Acid (DNA) was isolated from an individual larva using the QIAamp DNA Mini Kit (QIAGEN, Hilden, Germany). The larvae were identified to the species level using multiplex PCR, following the procedure outlined by the European Union Reference Laboratory for Parasites (European Union Reference Laboratory for Parasites, 2013).

## Ethical Approval and/or Informed Consent

Not applicable.

## Results

Using the SRPS EN ISO 18743:2016 method, the sample was determined to be positive for *Trichinella* spp. The burden of infection was 767 LPG. *T. britovi* was the only species identified after the multiplex PCR analysis of a total of 5 larvae.

**Fig. 2.**
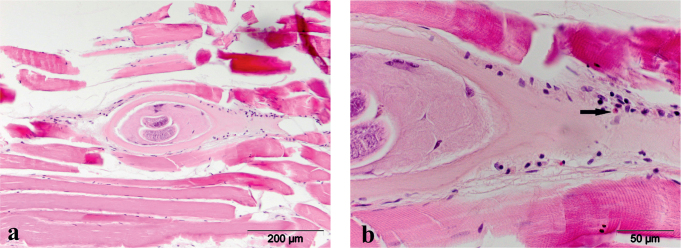
Histopathological changes in the wild boar diaphragm, HE a) encapsulated larva with perilarval inflamatory infiltrate, b) inflamatory infiltrate consists of mononuclear cells with individual eosinophills (arrow)

Histopathological examination revealed a large number of encapsulated *Trichinella* spp. larvae in the muscular tissue of the diaphragm. Mild inflammatory infiltrates, consisting primarily of mononuclear cells, lymphocytes, and macrophages, with individual eosinophils, were observed around the larvae (Fig. 2).

## Discussion

Trichinellosis is a zoonotic infection acquired by ingestion of infective *Trichinella* larvae. (Gottstein *et al*., 2009). Once ingested, these encysted larvae are released during digestion, where L1 larvae develop in the small intestine. The larvae then mature and produce newborn larvae, which migrate through various tissues in the body, leading to clinical manifestations of trichinellosis. Eventually, the larvae settle in striated muscle, where they become infective L1 larvae, completing their life cycle (Taratuto & Venturiello, 1997; Gottstein *et al*., 2009).

Despite the high larval burden, pathomorphological changes established by microscopic examination were slight, which corresponds to the literature data (Gamito-Santos *et al*., 2009).

Wild boar meat can carry numerous biological hazards transmissible to humans, including *Trichinella* spp. (Rostami *et al*., 2017). Therefore, the meat must be inspected for *Trichinella* infestation and properly cooked (Rostami *et al*., 2017). In 2016, a trichinellosis outbreak occurred in Serbia following the consumption of infected wild boar meat. Although the LPG was less than one, 116 people became infected (Dmitric *et al*., 2018). The LPG determined in this study was extremely high (767); therefore, we would like to emphasize the importance of meat inspection to reduce the risk that this hazard represents. High infections of *Trichinella* spp. have previously been detected in naturally infected wild boars (LPG 992) (Nöckler *et al*., 2006) and experimentally infected wild boars (LPG 1812, Germany) (Bessi *et al*., 2020); however, to our knowledge, this is the largest LPG so far detected in Serbia.
